# Validation of the “short health anxiety inventory” on a sample of school-age children (russian-language version)

**DOI:** 10.1192/j.eurpsy.2021.1367

**Published:** 2021-08-13

**Authors:** I. Shishkova, E. Pervichko

**Affiliations:** 1 Faculty Of Clinical Psychology, Ryazan State Medical University named after I.P. Pavlov, Ryazan, Russian Federation; 2 Faculty Of Psychology, Lomonosov Moscow State University, Moscow, Russian Federation; 3 Faculty Of Psychology And Social Sciences, Pirogov Russian National Research Medical University, Moscow, Russian Federation

**Keywords:** Short Health Anxiety Inventory, Health anxiety in children, Inventory validation

## Abstract

**Introduction:**

Modern Russian health psychology does not have the necessary tools for studying health anxiety in children, and therefore it is necessary to identify methods aimed at assessing the presence/absence and severity of children’s health anxiety.

**Objectives:**

To validate the “Short Health Anxiety Inventory” to a sample of school-age children who do not have serious physical disabilities.

**Methods:**

The sample: 193 respondents (average age-12.5; 117-girls). We used: “Short Health Anxiety Inventory” (SHAI; Salkovskis et al., 2002), Children CPQ (Factor C), “Attitude toward Health” questionnaire (Berezovskaya, 2005) (emotional scale), STAI (Spielberger, 2002), EPI (Eysenck, 1963) (neuroticism scale).

**Results:**

Correlation analysis suggests that “health anxiety” is a separate construct. The discriminativeness criterion shows that each individual statement, as well as the whole inventory, is aimed at measuring the same construct. The retest reliability assessment (4 weeks later) shows the results: the “Health Anxiety” scale - 0.892 (p≤0.01), the “Alertness to bodily sensations” scale - 0.889 (p≤0.01), the “Fear of negative consequences” scale - 0.815 (p≤0.01). Correlations between the scales shows the values: 0.943 (p≤0.01) - for the general scale, 0.392 (p≤0.01) - for the “Alertness to bodily sensations” scale, 0.675 (p≤0.01) - for the “Fear of negative consequences” scale. The original three-component structure of the questionnaire is confirmed. The Russian version of the inventory showed internal consistency (alfa-Cronbach’s coefficient - 0.835), retest reliability, discriminativeness, external and constructive validity.

**Conclusions:**

The results indicate that the SHAI can be used to study health anxiety in children due to its psychometric characteristics, simplicity and ease of use.

